# A QUALITATIVE ANALYSIS OF NARRATIVES CONTRIBUTED BY OLDER AFRICAN AMERICANS

**DOI:** 10.1093/geroni/igz038.2106

**Published:** 2019-11-08

**Authors:** David Schlundt, Kemberlee R Bonnet

**Affiliations:** vanderbilt University, Nashville, Tennessee, United States

## Abstract

We analyzed 19 interviews of African Americans who came of age during desegregation. We approached the development of the coding system and analysis of coded transcripts using an iterative inductive/deductive approach guided theoretically by the social ecological framework and life course development theory. Major coding categories included time orientation, topics of discussion, emotions, habits and behaviors, family, community, race relations, and history. Analysis of the sorted and coded quotes led to the development of a conceptual framework for understanding how participants portrayed their life trajectories. Individuals’ lives occurred in the context of societal institutions, the larger society, and culture. Life trajectories involve a series of changes in life circumstances and social relationships during which individuals engage with life goals such as making a living, staying healthy, coping with stressors, and maintaining satisfactory interpersonal relationships. Important themes associated with healthy aging included resilience, faith, forgiveness, and family.

